# Acousto-Optic Q-Switched Fiber Laser-Based Intra-Cavity Photoacoustic Spectroscopy for Trace Gas Detection

**DOI:** 10.3390/s18010042

**Published:** 2017-12-25

**Authors:** Qinduan Zhang, Jun Chang, Qiang Wang, Zongliang Wang, Fupeng Wang, Zengguang Qin

**Affiliations:** 1 School of Information Science and Engineering and Shandong Provincial Key Laboratory of Laser Technology and Application, Shandong University, Jinan 250100, China; zhang_qinduan@163.com (Q.Z.); fp.wang1990@gmail.com (F.W.); qinzengguang@sdu.edu.cn (Z.Q.); 2 Department of Mechanical and Automation Engineering, The Chinese University of Hong Kong, New Territories, Hong Kong, China; woshiwq1989@163.com; 3 School of Physics Science and Information Technology and Shandong Key Laboratory of Optical Communication Science and Technology, Liaocheng University, Liaocheng 252059, China; wangzliang888@163.com

**Keywords:** acousto-optic modulator, Q-switching, photoacoustic spectroscopy, J0101

## Abstract

We proposed a new method for gas detection in photoacoustic spectroscopy based on acousto-optic Q-switched fiber laser by merging a transmission PAS cell (resonant frequency *f*_0_ = 5.3 kHz) inside the fiber laser cavity. The Q-switching was achieved by an acousto-optic modulator, achieving a peak pulse power of ~679 mW in the case of the acousto-optic modulation signal with an optimized duty ratio of 10%. We used a custom-made fiber Bragg grating with a central wavelength of 1530.37 nm (the absorption peak of C_2_H_2_) to select the laser wavelength. The system achieved a linear response (R^2^ = 0.9941) in a concentration range from 400 to 7000 ppmv, and the minimum detection limit compared to that of a conventional intensity modulation system was enhanced by 94.2 times.

## 1. Introduction

Photoacoustic spectroscopy (PAS) is a common and promising technique for trace gas analysis. After its appearance, PAS did not obtain substantial progress in a very long period until the invention of laser. Since then, PAS has seen significant development in both theory and implementation with the help of CO and CO_2_ laser [1,2], quantum cascade laser (QCL) [3,4], and fiber laser [5] which are used for gas detection.

According to the Beer–Lambert law [6], when a bunch of monochromatic infrared lasers pass through a gas medium, photons are absorbed by the gas molecules, leading to intensity attenuation. The intensity change can be described by the following equation [7], I(ν) = I_0_(ν)exp[−α(ν)*CL*] = I_0_(ν)[1 − α(ν)*CL*], where I_0_(ν) is the incident laser power, I(ν) is the transmitted laser power, α(ν) is the gas absorption coefficient and *C* is the gas concentration. So, the absorbed laser power I_a_(ν) as I_a_(ν) = I_0_(ν)α(ν)*CL*. The detected PAS signal *S* is related to the absorbed laser power I_a_(ν) as *S* ∝ I_a_(ν)·*Q*/*f*_0_, where *Q* and *f*_0_ are the quality factor and resonant frequency of the acoustic resonator respectively. The absorbed laser power is directly proportional to the PAS signal. Hence, the PAS signal is directly proportional to the incident laser power. The linear relationship between PAS signal amplitude and incident laser power provides an attractive feature that the sensitivity of PAS-based sensors benefits from high-power laser [8].

To increase the laser power, one typical way is to utilize the external enhanced optical cavity for power buildup. Rossi et al. [9] introduced an optical enhancement method applied to a diode laser photoacoustic trace gas detector, the laser intensity inside the acoustic resonator is amplified using a Fabry–Pérot cavity. Tens to hundreds ppbv-level sensibility for molecules absorbing can be obtained. Wojtas et al. [10] recently reported a mid-infrared nitric oxide (NO) detection using intra-cavity quartz-enhanced photoacoustic spectroscopy (I-QEPAS) by a compact, high-finesse bow-tie optical cavity with an integrated resonant quartz tuning fork (QTF). As a result, a minimum detection limit (MDL) of 4.8 ppbv at a 30 ms integration time was achieved. However, all these external optical cavities require careful optical design and sophisticated optical feedback-locking techniques. Another way to improve the sensitivity of PAS-based sensors is to employ an optical amplifier to boost the laser power of a tunable seed laser. Ma et al. [8] employed an erbium-doped fiber amplifier (EDFA) to amplify distributed feedback diode laser with a central wavelength of 1.53 μm, which was used as the excitation source, and achieved a 33.2 ppbv MDL. Optical power inside the laser cavity is normally much higher than the laser output, indicating a promising approach for highly sensitive PAS detection. Wang et al. [11] reported fiber-ring laser-based intra-cavity PAS (FLI-PAS) for trace gas sensing to utilize the high intra-cavity laser power and a maximum intra-cavity laser power of ~108 mW in the FLI-PAS based sensor was estimated. They achieved a good linear response (R^2^ = 0.996) to C_2_H_2_ concentration and a MDL of 390 ppbv at 2-s response time.

It is well known that Q-switching of fiber laser is a suitable technique to obtain high-power pulsed laser. Q-switch periodically adjusts the laser cavity loss, which stores a large amount of energy at high loss and releases energy at low loss. Delgado-Pinar et al. [12] reported actively Q-switching of an all-fiber laser using a Bragg grating based acousto-optic modulator, achieving a peak pulse power of 10 W, and a 82 ns pulse width was generated. Shi et al. [13] proposed a master oscillator power amplifier (MOPA)-based pulsed fiber laser and achieved a peak pulse power of >63 kW for transform-limited fiber laser pulses at ~2 μm regime. Zhang et al. [14] reported a Q-switched fiber MOPA oscillated at 1064 nm. The pulse duration is about 157 ns corresponding to the peak pulse power of 37.1 kW. Recently, He et al. [15] demonstrated a high peak pulse power, short-pulse, Q-switched Yb-doped large-mode-area photonic crystal fiber (PCF) oscillator, producing a peak pulse power of 130 kW at 10 kHz. The Q-switched fiber lasers not only have the advantage of high power, but also have a wide range of output wavelength, compact structure, and are compatible with fiber for optical sensing.

Piao et al. [16] presented a nanosecond Q-switched erbium-doped fiber laser (EDFL) and the laser was tested to generate the photoacoustic signal from a human hair. Aytac-Kipergil et al. [17] reported a unique fiber laser developed specifically for multiwavelength photoacoustic microscopy system. For both sensing systems, only part of the laser power was split from the laser cavity by a fiber coupler for acoustic wave excitation, and the high intra-cavity power is of great potential to be exploited for enhanced PAS signal.

In this letter, an acousto-optic Q-switched fiber laser-based intra-cavity photoacoustic spectroscopy was proposed for trace gas detection. A maximum peak pulse power of ~679 mW was generated by the acousto-optic Q-switched fiber laser with an optimized duty ratio of 10% selected for the modulation signal. The pulse power is about 6.3 times as much as that reported in [11]. Our current gas detection system is expected to obtain higher power by optimizing the power of pump source and the characteristics of erbium doped fiber (EDF). A custom-made PAS cell with a resonant frequency of 5.3 kHz was placed inside the fiber ring cavity to generate and accumulate the photoacoustic wave, which was then detected by a microphone. C_2_H_2_ was chosen as the analyte due to its important applications in the detection of fault gases in transformers [18] and in ethylene streams for polyethylene production [19]. The acousto-optic Q-switched system achieved a good linear response (R^2^ = 0.9941) in a concentration range from 400 to 7000 ppmv and a minimum detectable (C_2_H_2_) gas concentration of 8.4 ppmv. The MDL compared to that of a conventional intensity modulation system was enhanced by 94.2 times.

## 2. Experiment Setups

**Figure 1:**
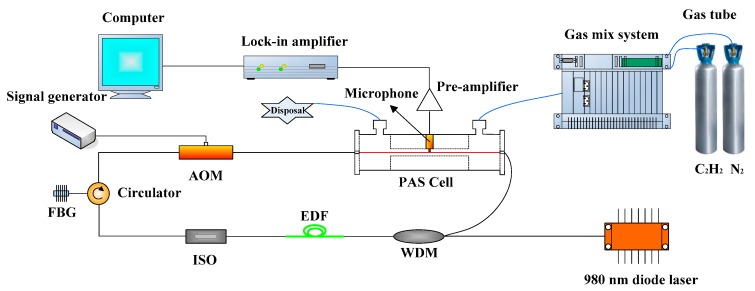
Schematic of the PAS system with acousto-optic Q-switched fiber laser.

**Figure 2:**
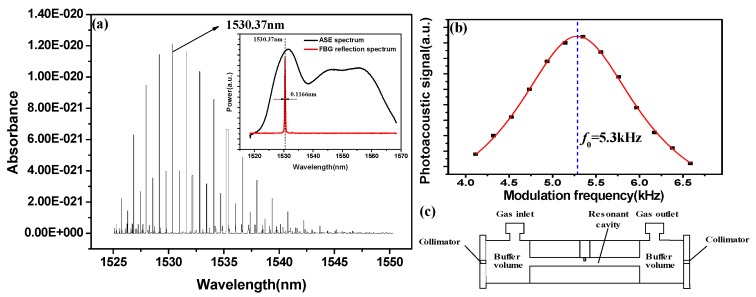
(**a**) C_2_H_2_ absorption lines in C band. Inset: ASE spectrum and reflection spectrum of the custom-made FBG; (**b**) Resonance profile of the PAS cell: resonant frequency, 5300 Hz; (**c**) Schematic of the PAS cell.

A schematic diagram of the PAS system with acousto-optic Q-switched fiber laser is shown in Figure 1. The pump power of the 980-nm diode laser (S26-7602-140, JDSU(SDL), Vancouver, BC, Canada) is 80 mW. A 980/1550 wavelength division multiplexer (WDM) was implemented to combine the 980-nm pump laser and the intra-cavity near-infrared laser into the fiber-ring cavity. A 20-m long EDF (MP980, OFS, Norcross, GA, USA) with a doping concentration of 1500 ppm and an absorption parameter of 6 dB/m at 1530 nm was used as the gain medium. After being forward pumped by 980-nm laser, the EDF could generate a wide amplified spontaneous emission (ASE) spectrum, covering 1520–1570 nm. The isolator (ISO) was used to force the laser propagation in a single direction, the circulator with direction selectivity ensured the unidirectional laser propagation inside the ring cavity. A custom-made fiber Bragg grating (FBG) with a bandwidth (3 dB) of 116.6 pm and a Bragg wavelength of 1530.37 nm was used as a wavelength selector in this EDFL system, determining its output wavelength. To prevent ambient-based wavelength shift, a necessary strategy was adopted to control the FBG temperature. Figure 2a presents the C_2_H_2_ absorption lines in C band and shows a strong absorption peak at 1530.37 nm [20]. The inset graph shows the ASE spectrum and the reflection spectrum of the FBG with the irradiation of the ASE light source.

The output of the circulator was connected to an acousto-optic modulator (AOM) (T-M200-0.1C2J-3-F2P, Gooch&Housego, Ilminster, UK). Modulation signal was provided by a signal generator (FY2300A, Feel Tech, Zhengzhou, China) to control the AOM. The modulation frequency of AOM is 5.3 kHz, which is consistent with resonant frequency of PAS cell. When the AOM is closed, the laser is in a low-Q state to accumulate enough population inversion for the successive pulse. When the AOM is opened, the laser pulse is released. The cavity loss of the laser is periodically adjusted by the opening and closing of the AOM to obtain laser pulses. At the initial time, the laser resonator is in the low-Q state with high loss, and the density of the inversion population is continuously accumulated. After that, the laser resonator is in the high Q-value state with low loss, and the accumulated inversion population is instantly released. Thus, a Q-switched laser pulse with narrow pulse width and high peak power is formed.

The custom-made transmission PAS cell (as shown in Figure 2c) with an insertion loss of ~0.8 dB was placed inside the fiber ring cavity. The PAS cell includes a cylindrical channel with a length of 34 mm and a diameter of 6.4 mm, performing as an acoustic resonant cavity. The standing acoustic wave can be formed in the acoustic resonant cavity where the excitation laser beam is located. An electret condenser microphone (EK-3024, Knowles, Itasca, IL, USA) is placed at the middle of the channel to detect the acoustic wave, and it can convert acoustic wave into current signal. Two 17 mm long buffer volumes were constructed at both ends of the acoustic resonant cavity. The beam is easily collimated because the distance between the two collimators is 68 mm and the diameter of the acoustic resonant cavity is 6.4 mm. However, to reduce the external interference, the tightness of PAS needs to be improved. Figure 2b shows the frequency response of the PAS cell. PAS cells with different resonant frequency can be designed by properly selecting the length and diameter of the acoustic resonant cavity. The current signal is converted to a voltage signal by a pre-amplifier chip (CA3140E, Intersil, Milpitas, CA, USA). A lock-in amplifier (Model 7230, AMETEK, Berwyn, PA, USA) is used to extract the harmonic signals and the computer is used to display the signals. Samples with different C_2_H_2_ concentrations are generated by a gas mix system (RCS 2000-A, Beijing Kingsun Electronics, Bejing, China) by mixing different proportions of pure N_2_ and 1% C_2_H_2_.

## 3. Experiment Results and Discussion

**Figure 3:**
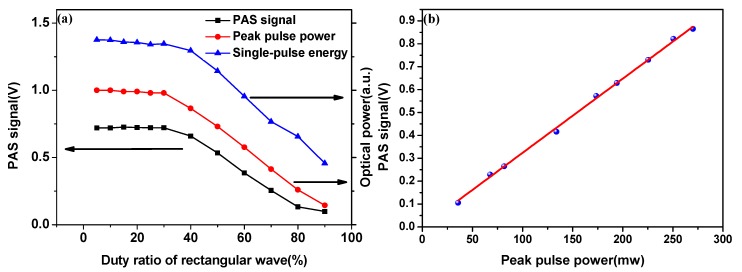
(**a**) Measured PAS signal, peak pulse power, and single-pulse energy at different duty ratio of modulation signal; (**b**) Response of PAS signal to peak pulse power in the case of a constant average power.

With the modulation frequency of AOM set as 5.3 kHz, the relation between the optical power and the duty ratio of the modulation signal is shown in Figure 3a. The optical power and duty ratio are monitored by using an optical power meter and an oscilloscope, respectively. The peak power of the pulse and the single-pulse energy hold relatively stable within duty ratio of 30%, then the peak power of the pulse and the single-pulse energy decrease with the increase of the duty ratio. Because the closing time of the AOM decreases gradually, the number of the inversion population accumulated on the upper level of Er^3+^ decreases gradually and the Q-switched pulse does not have enough time to build up into a full Q-switched pulse. So that the output energy is reduced after the AOM is opened. Meanwhile, PAS signal is monitored, and it shows the same tendency as the optical power. In this work, a duty ratio of 10% was selected for the modulation signal to maximize the PAS signal.

To further verify the effect of optical power on the PAS signal, we carried out the following experiments using an external cavity gas detector. Average power can change with different modulation signal duty ratio, and the average power is maintained with the assistant of a tunable attenuator in the following experiment. Thus, the energy should remain the same in every cycle in the case of constant modulation frequency. Figure 3b shows that the PAS signal increases with the peak pulse power.

**Figure 4:**
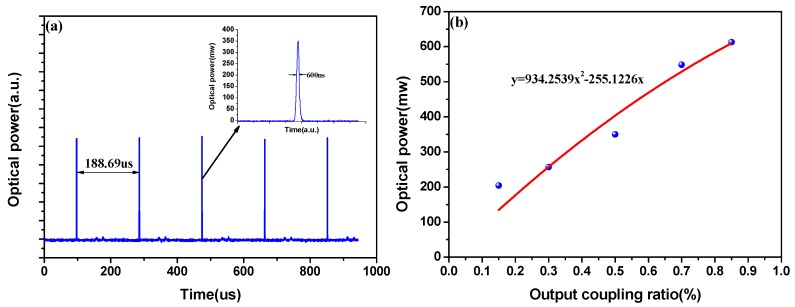
(**a**) Measured Q-switched pulse sequence at modulation frequency of 5.3 kHz. Inset: Typical Q-switched pulse at modulation frequency of 5.3 kHz; (**b**) Measured fiber laser output power at different output coupling ratios.

Figure 4a depicts the pulse sequence of the laser when the modulation frequency is 5.3 kHz, and their intervals are about 188.69 μs. The waveform of the laser output pulse from the oscilloscope is shown in the inset graph of Figure 4a, indicating a pulse width of ~600 ns.

To estimate the maximum peak pulse power in the cavity, a 1 × 2 coupler was used to split the intra-cavity laser power for monitoring. Note that this coupler should not be included in practical applications to minimize unnecessary optical losses. Figure 4b depicts the output peak pulse power measured with an oscilloscope. The output peak pulse power was successively measured using five single-mode fiber couplers with the different output coupling ratio. The intra-cavity peak pulse power was estimated using the known coupling ratio of the fiber coupler and the measured output peak pulse power. As shown in Figure 4b, the output peak pulse power increases with the coupling ratio. Conversely, the highest intra-cavity peak pulse power can be obtained by reducing the output coupling ratio of the fiber coupler. A maximum intra-cavity peak pulse power was evaluated to be ~679 mW, corresponding to the case when the output coupler is not used in the sensing system.

**Figure 5:**
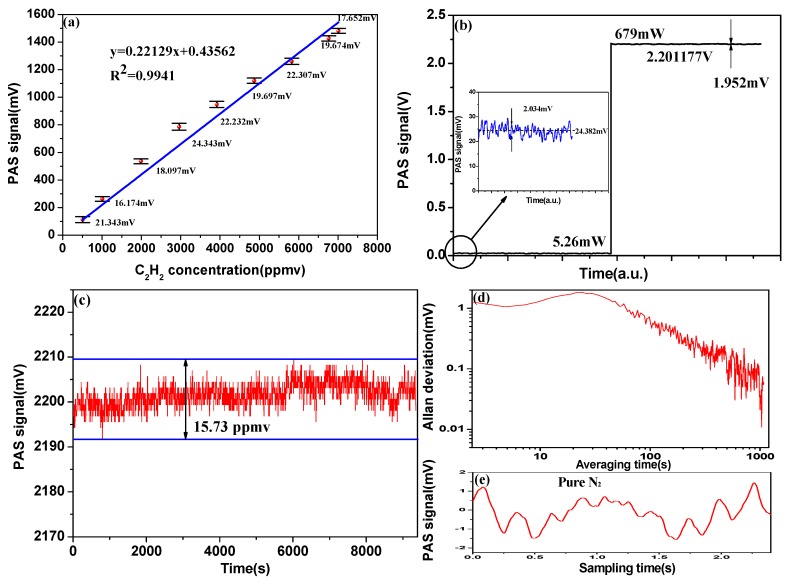
(**a**) Linearity of the PAS signal to the C_2_H_2_ concentration at 1 bar and room temperature (24 °C); (**b**) The PAS signal in conventional intensity modulation system and acousto-optic Q-switched system; (**c**) Long-term stability of acousto-optic Q-switched system for 9500-ppmv C_2_H_2_; (**d**) Allan deviation analysis of the acousto-optic Q-switched system as a function of integration time, the measurement was carried out with the PAS cell filled with pure N_2_; (**e**) The representative PAS signals for pure N_2_.

Linearity experiments are performed using the PAS system. Several C_2_H_2_ samples with different concentrations were generated by a gas mix system and introduced into the PAS cell with a flow rate of 500 mL/min. All the measurements were performed at a pressure of 1 bar and room temperature (24 °C). Figure 5a depicts the linear response of the PAS system. A linear fit to the experimental data yields an R^2^-value of 0.9941, indicating a good linear response of the sensor to C_2_H_2_ concentration. The error bars in the vertical axis show the accuracy and reproducibility of the measurement and the corresponding magnitudes are listed.

We compared the acousto-optic Q-switched system with a conventional intensity modulation system. A gas mix system was used to produce 9500 ppmv C_2_H_2_ sample. Figure 5b depicts a MDL of 791 ppmv when the power of the conventional intensity modulation system is 5.26 mW. In the conventional intensity modulation system, we placed the gas detection system outside the laser cavity, and used a semiconductor laser instead of the fiber laser. The intensity of the laser is modulated by the AOM to achieve intensity modulation. The modulated laser is absorbed by the gas molecules to produce acoustic wave, and the acoustic wave is detected by the microphone to produce a PAS signal. For the acousto-optic Q-switched system, with the standard deviation measured to be 1.952 mV, the MDL is estimated to be 8.4 ppmv. The MDL compared to that of a conventional intensity modulation system was enhanced by 94.2 times. The NNEA is estimated to be 7.33 × 10^−9^ W cm^−1^ Hz^−1/2^. A long-term detection experiment is performed at a pressure of 1 bar and room temperature to test the long-term stability of the acousto-optic Q-switched system. As shown in Figure 5c, the maximum signal fluctuation for 9500 ppmv C_2_H_2_ is measured to be 15.73 ppmv. This may be due to the temperature-based drift of the FBG. In order to eliminate the influence of stochastic noise on PAS signal, we used the average algorithm to deal with the PAS signal. For evaluating the acousto-optic Q-switched system, we performed an Allan variance [21] analysis, measuring and averaging the PAS signal at zero C_2_H_2_ concentration (pure N_2_) for about three hours, as shown in Figure 5d. The figure indicates that the system allows data averaging without baseline or sensitivity drift on a more than 1000 s time scale. Figure 5e depicts the measured representative PAS signals for pure N_2_, and shows the noise features of the measurements related to the laser and PAS cell.

## 4. Conclusions

In this paper, acousto-optic Q-switched photoacoustic spectroscopy was proposed for gas detection with a PAS cell placed in fiber laser cavity, and the high-power pulses are used for acoustic wave excitation. A peak pulse power of ~679 mW was obtained using an acousto-optic modulation signal with an optimized duty ratio of 10%. The sensor achieved a good linear response (R^2^ = 0.9941) in concentration range from 400 to 7000 ppmv, and a MDL of 8.4 ppmv, corresponding to a NNEA of 7.33 × 10^−9^ W·cm^−1^·Hz^−1/2^. The MDL compared to that of a conventional intensity modulation system was enhanced by 94.2 times. Due to the system using a PAS cell with poor sealing performance, it is easy to be influenced by external noise and gas flow, and the noise of the home-made pre-amplifier circuit has a bad influence on the PAS signal. With Allan variance analysis, we can optimize the performance of the system by increasing the average number of PAS signals. In the future work, we will redesign the PAS cell and pre-amplifier circuit. We also have to reduce the loss of optical devices as much as possible, such as the circulator, isolator, and other loss components. We can also increase the peak pulse power by increasing the power of the pump source [22]. Sub-ppmv, even ppbv level, of sensitivity can be expected after optimization. In this work, we chose EDF as the gain medium, whose spectral range can be extended to 6200–6550 cm^−1^ [23]. This particular spectral range covers absoption lines of several important gas specises such as NH_3_ and CO. Hence, this technique can be readily employed to detect more gas specises by properly choosing the Bragg wavelength of the FBG, which acts as a wavelength selector in the EDFL.
